# High resolution, week-long, locomotion time series from Japanese quail in a home-box environment

**DOI:** 10.1038/sdata.2016.36

**Published:** 2016-06-07

**Authors:** Diego A. Guzmán, Stefania Pellegrini, Ana G. Flesia, Miguel A. Aon, Raúl H. Marin, Jackelyn M. Kembro

**Affiliations:** 1 Instituto de Investigaciones Biológicas y Tecnológicas (IIByT, CONICET), and Instituto de Ciencia y Tecnología de los Alimentos, Cátedra de Química Biológica, Facultad de Ciencias Exactas, Físicas y Naturales, Universidad Nacional de Córdoba, 1611 Vélez Sarsfield, X5016GCA, Córdoba, Argentina; 2 Department of Animal Science, Aarhus University, 20 Blichers Allé, Post Box 50, DK-8830, Tjele, Denmark; 3 Centro de Investigaciones y Estudios de Matemática (CIEM, CONICET), and Facultad de Matemática, Astronomía y Física FAMAF, Universidad Nacional de Córdoba, Ing. Medina Allende s/n Ciudad Universitaria, Córdoba, CP X5000HUA, Argentina; 4 Johns Hopkins University, School of Medicine, Baltimore, Maryland 21205, USA

**Keywords:** Data publication and archiving, Time series, Animal behaviour, Power law, Scale invariance

## Abstract

Temporal and spatial patterns of locomotion reflect both resting periods and the movement from one place to another to satisfy physiological and behavioural needs. Locomotion is studied in diverse areas of biology such as chronobiology and physiology, as well as in biomathematics. Herein, the locomotion of 24 visually-isolated Japanese quails in their home-box environment was recorded continuously over a 6.5 days at a 0.5 s sampling rate. Three time series are presented for each bird: (1) locomotor activity, (2) distance ambulated, and (3) zone of the box where the bird is located. These high resolution, week-long, time series consisting of 1.07×10^6^ data points represent, to our knowledge, a unique data set in animal behavior, and are publically available on FigShare. The data obtained can be used for analyzing dynamic changes of daily or several day locomotion patterns, or for comparison with existing or future data sets or mathematical models across different taxa.

## Background & Summary

Locomotion, movement through the environment^1^, is not an end in itself but a means to attain an end, like finding food, a mate, a place of refuge from adverse environmental conditions or from enemies^2^, or a suitable nesting site. As a result, temporal and spatial patterns of locomotion reflect both resting periods, as well as the necessity to move from one point to another to satisfy physiological and behavioural needs. Characteristic 24 h circadian rhythms of sleep/wake cycles are found in many animal species^3–5^. Ultradian rhythms with periods less than 24 h have also been observed in locomotion^6,7^. Interestingly, ultradian behavioural rhythms are often related to feeding behaviour^8^. For example, individually housed quails exhibit ultradian feeding rhythms of approximately 45 min^9^, which help explain ultradian rhythms in locomotion with a similar period^10^.

Execution of appropriate motor responses to stimuli is essential for animal survival representing one of the most fundamental aspects of nervous system function^11^. Although the basic principle of locomotion appears simple, motor behaviour emerges from interactions between the animal’s muscular, skeletal, nervous, respiratory, and circulatory systems, and the environment^1,12^. From these interactions complex temporal dynamics emerge.

Locomotion has been studied in diverse areas of biology and biomathematics. In chronobiology, locomotion has been widely used as a convenient non-intrusive way to measure circadian and ultradian rhythms^13^. For biologists, locomotor extremes are attractive because they provide clear examples from which to determine structure-function relations^1^. In addition to rhythmic behaviour, locomotor activity exhibits robust scale-invariant fractal patterns in dynamics (i.e. patterns composed of parts that resemble the whole at different magnification scales) and long-range temporal correlations (i.e. the process is characterized by infinite memory, where the autocorrelation function decays according to a power-law^14^)^15–18^. The scaling and self-similarity traits of fractals reflect the intrinsic interdependence among the different functional levels of organization exhibited by living systems, and represent an emergent property from underlying multi-scale physiological processes and their environmental interactions^7^. Specifically, it has been proposed that the fractal dynamics of animal locomotion is regulated by both the central and the intra spinal components of the nervous system, involving feedback loops, a central pattern generator^19–21^, psychophysical control^22^, and intrinsic activity control mechanisms^23^.

**Figure 1:**
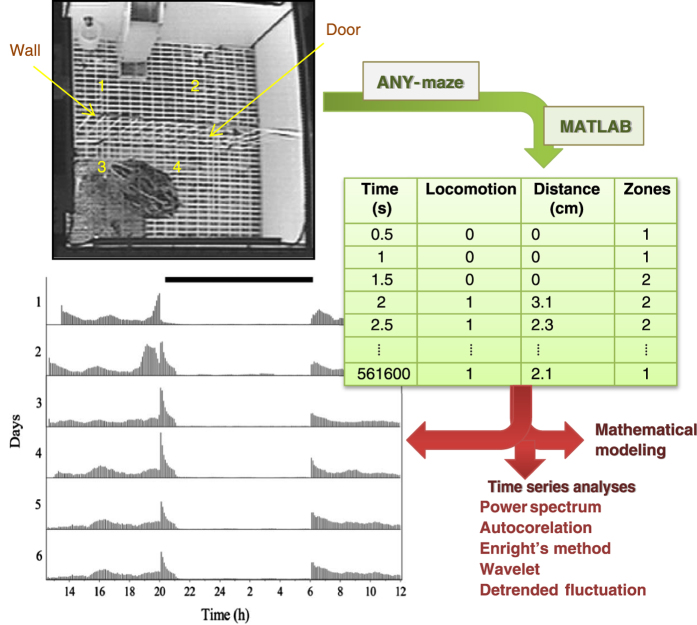
Workflow diagram of the experimental procedure. Quail locomotor behavior in a home-box environment was continuously recorded during 6.5 d with a computer and the video recordings processed offline using ANY-maze and MATLAB. The experimental box had a wire-mesh floor, and a metal bar wall that divided the box into two same-size compartments (feeding, nesting). The metal bar wall had an opening that served as a door allowing the bird to move freely between compartments. The box was imaginarily divided into four zones (1–4). In zone 1 a feeder and an automatic nipple drinker were positioned, and in zone 3 a small rubber nest was placed. Three time series at 0.5 s intervals were obtained for each animal: (I) locomotor (a zero or a one was recorded when the animal was immobile or mobile, respectively); (II) distance ambulated; and (III) zone location (denoting the location of the animal centre during the time interval). Locomotor time series were used to construct actograms^10^ and to perform time series analysis^10^.

Mathematically speaking, fractals in their infinite but reducible complexity can be considered opposites to smooth oscillatory rhythms. The simultaneous presence of rhythmic and fractal patterns in our time series of quails’ locomotor behaviour demanded high-resolution, long-time series that are necessary for characterizing fractal dynamics and rhythms detection over a range of time scales from seconds to a day. Therefore, to evaluate in our study whether any link exists between ultradian rhythms and the fractal dynamics observed, we recorded time series of locomotion in visually isolated female Japanese quails sampled every 0.5 s during 6.5 days (>10^6^ data points)^10^. From each animal we obtained three time series: (1) locomotor, (2) distance ambulated, and (3) zone location (Fig. 1). These long, high-resolution time series consisting of 1.07×10^6^ data points represent, to our knowledge, a unique data set in animal behavior. Elsewhere^10^ we analyzed circadian and ultradian rhythms using the locomotor time series from this study.

Most studies of fractal locomotor temporal dynamics have focused on mammals, principally rodents and humans^7,15,23–25^. In mammals, the suprachiasmatic nucleus (SCN) is a critical node involved in the generation of both circadian rhythmicity and fractal dynamics^7^. Although similarities in the temporal dynamics of mammals and birds locomotion have been reported^10^, avian species do not possess SCN. Instead, it has been hypothesized that circadian pacemaking in birds involves at least three autonomous and anatomically distinct oscillators- retina, pineal gland, and hypothalamus^26^. The predominant walking mode of the Japanese quail provides an avian animal model for comparing with mammal locomotor dynamics. In this framework, the week-long time series studied herein could be used to for comparison with existing or future data sets, and mathematical models developed in other species.

## Methods

Herein, we describe in more detail the methods employed, and describe the distance and zone location time series that were not analysed previously^10^.

All the procedures were in compliance with the *Guide for the Care and Use of Laboratory Animals* issued by the National Institute of Health (NIH Publications, Eighth Edition). The experimental protocol was approved by the Institutional Committee for the Care and Use of Laboratory Animals (Comité Institucional para el Cuidado y Uso de Animales de Laboratorio (CICUAL)) of the Facultad de Ciencias Exactas, Físicas y Naturales (FCEFyN)—Universidad Nacional de Cordoba, Argentina.

### Animals and housing

Japanese quail (*Coturnix japonica*) eggs were collected from parent birds housed in the facilities of FCEFyN—Universidad Nacional de Cordoba, Argentina. Eighty mixed-sex Japanese quail hatchlings were randomly housed in 2 white wooden boxes (40 quail each) measuring 90×80×60 cm (length×width×height) with a feeder along one wall, and 14 automatic nipple drinkers. At 28 days of age, female quail were randomly cage-housed in pairs in a 6-tier cage battery units, where each cage measured 40×20×21 cm (length×width×height), equipped with a feeder covering the front of the cage and an automatic nipple drinker^27^. Quails were subjected to a daily cycle of 14 h light (300 to 320 lx):10 h dark during the study, with lights on from 6am to 8pm. Feed and tap water was provided *ad libitum*. These experimental conditions were maintained throughout the experiment. Due to management practices females were exposed to a brief (5 min) male-female sexual interactions up to 10 days prior to testing. All animals used in experiments were examined and showed no signs of wing-fracture or other lesions.

### General procedure

The experimental setup consisted of a white wooden box measuring 40×40×40 cm (width×length×height, respectively) with a white wire-mesh floor (1 cm grid to allow the passage of excreta). The floor was raised 12 cm from the bottom of the box. This distance made the excreta remain out of focus (blurred) in the video recording, therefore a uniform background was achieved throughout the study (an important feature for the video tracking system). A metal bar wall (2.5 cm separation between bars) divided the box in two same-size compartments (feeding, nesting), and a 7.5×7.5 cm opening served as a door allowing the bird to move freely between compartments. In the feeding compartment, a feeder and an automatic nipple drinker (with drainage) were positioned, while in the nesting compartment, a small white rubber nest measuring 15×15 cm^28^ was placed (Fig. 1). Each of these two compartments were then imaginarily divided into two zones, resulting in a total of 4 zones of equal dimensions for data analysis (see below and Table 1 for more details). Nylon monofilament line was extended over the top of the boxes with a 1 cm separation in order to prevent the birds from potential escape attempts without interfering with their visualization from top. To achieve uniform illumination and reduce shadow projection of the bird around the upper perimeter of the box, LED (Light-Emitting Diodes) were installed, the resulting light intensity was similar to rearing conditions. A video camera was suspended 1.5 m above the box. These cameras have built-in infrared LED lighting, automatically switching to infrared recording after lights were turned off.

The locomotor activity of 24 fully adult female quail (100-140 days old) was evaluated. Testing began at approximately 1:00 PM, when 12 quail were placed in a box, and transported to a nearby experimental room. Each bird was then placed individually in the feeding compartment in one of the 12 boxes. During the following 6.5 days, the birds’ ambulatory activity was continuously recorded onto a computer that was accessed remotely by experimenters. This arrangement made certain that the experimenters did not disturb the birds during testing. Testing ended at 9 PM on the 7th day. Daily maintenance activities (egg collection, cleaning and feeding) were performed every day between 12 and 12:30. Due to interference of experimenters in the recording of behaviour, the locomotor activity during that period was not included in time series. In total this implied a loss of <2% of information from the data set and therefore it is not considered that overall analyses are significantly affected^29^. Since only 12 birds could be tested simultaneously, two consecutive batches (groups) of 12 individuals were evaluated. This sample size selected allowed evaluation of the variability between conspecifics and of the reproducibility of experiment by comparing two independent batches.

We used the ANY-maze Video Tracking System software computer program to register the birds’ movement and location at 0.5 s intervals. Thus, for each bird, three time series (locomotor, distance, and zone location) consisting of 1.07×10^6^ time intervals, were obtained from the 6.5 day test period (Fig. 1). ANY-maze uses dynamic subtraction as the detection method to locate and track the animals. Using this standardized tracking technique, the software renders a series of pixels in a given frame (considered to be the animal), comparing thereafter these pixels from one frame with those in the next, and evaluates which percentage of them are common. If the percentage of common pixels is greater than the value of the parameter ‘*immobility sensitivity (%)*’ then the animal is considered to be immobile. If the animal is immobile, the older frame is retained and used as a reference for comparing with the next frame. If immobility lasts longer than the ‘*minimum immobility period*’, then ANY-maze reports the animal as immobile, with the bout initiated at the start of the period. In our experiment, the animals appear darker than the white background of the apparatus. The parameter ‘*immobility sensitivity (%)*’ was determined empirically as 70%, while the second parameter ‘*minimum immobility period*’ was determined as 0.5 s given that it represents, approximately, the minimum duration of an actual quail step.

#### Locomotor time series

Behavioural data was recorded in the form of a time series of mutually exclusive states: mobile/immobile. At any given time, if the bird was moving a number one was recorded or a zero if immobile.

#### Distance time series

From the x,y coordinates of the position of the centre of the animal at each time interval (*t*), the distance (D) moved by the centre of the animal was computed as D(*t*)=√((x(*t*)−x(*t−*1))^2^+(y(*t*)−y(*t−*1))^2^), then transformed from pixel to cm.

#### Zone location time series

In the experimental box, four zones that measure approximately 20×20 cm were designated, and arbitrarily assigned a number one through four (Fig. 1). A brief description of each zone is provided in Table 1. At each time interval, the number of the zone where the centre of the bird was located was recorded.

As a caveat, although the ANY-maze software registers the behavioural data at 0.5 s intervals, during prolonged periods of inactivity where the coordinates of the animal do not change, the ANY-maze software does not write this redundant information, thus shortening the time series which look slightly shorter than the actual time series. Therefore, the missing regions of data from the ANY-maze data series were inspected using a simple Matlab code to adjust for a constant sampling interval throughout the time series^30^.

### Code availability

Custom made code for correcting ANY-maze time series (see Methods Section^30^) and for data analysis (see Usage Note Section) using Wavelets^31^ and Detrended Fluctuation Analyses^32^ can be downloaded from FigShare.

## Data Records

All time series from this study are stored in the public repository FigShare as individual text files (.txt). For practical purpose, data from each time of time series were grouped in file sets according to time series type (locomotor, distance or zone location; see Methods section for details) (Data Citation 1, Data Citation 2 and Data Citation 3). Table 2 provides reference to each file set and examples of data file names. Considering that the 24 animals were evaluated in 2 consecutive experimental batches (groups) of 12 birds, each subject quail of a given batch was arbitrarily assigned a number one through twelve. In the file name an indication of the corresponding bird is provided as ‘Quail_XX_group X’. In addition for distance and zone time series, references to time series type are also noted in the file name.

## Technical Validation

Data sets were collected using the commercially available ANY-maze^TM^ Video Tracking System software (www.anymaze.com). This software is a flexible video tracking system designed for automated testing of behavioural experiments such as those described herein. In our laboratory, we have used this software previously in order to obtain locomotor time series^16,27^. One of the advantages of ANY-maze is that the researcher can perform visual observation of the tracking being performed and the output being recorded. When a video record of the experiment is being analyzed, both the centre of the animal and their body is highlighted, also text indicates whether the animal is moving or not, and the zone where the animal is located appears on the screen. In order to validate the correct tracking of the animal, visual observations of tracking being performed by ANY-maze was conducted during 10 min intervals at least every 6 h of recording for all 24 animals. The high contrast between the white, well illuminated, box and the dark brown quail feathers enabled an accurate tracking of the animal.

## Usage Notes

Locomotor time series were analyzed using an array of tools including Actograms, Power Spectrum Analysis, Autocorrelation, Enrights’ method, Wavelets and Detrended Fluctuation Analysis^10^. The customized code utilized for Wavelets^31^ and Detrended Fluctuation Analyses^32^ can be downloaded from Figshare. These two MATLAB codes are written as a function (wavelet_agf.m and dfa_jmk.m, respectively) and are briefly described below.

The first routine runs wavelet analysis on a time series (TimeSeries) with the complex Morlet wavelet, calling the cwt function of Wavelet Toolbox of Matlab. As an output this routine shows plots of the real, imaginary, modulus and phase angle of the wavelet coefficients for time scales between 0 and 33 h. This program was designed for analyzing time series obtained at a sampling rate <6 min, thus as an input sampling rate expressed in seconds must be provided (sampling_rate). For example, for this experiment a sampling rate of 0.5 s was used. The wavelet analysis will be performed on data binned every 6 min (SERIES_6 min), in order to reduce noise. In addition, to correctly plot data the time expressed in hours that the data was obtained should be provided (TimeHours). To run the function write in the Command Window of MATLAB the command: [c_6 min period_6 min SERIE_6 min Time6 min]=wavelet_agf (TimeHours,TimeSeries,sampling_rate).

The second function can be used for performing Detrended Fluctuation Analysis on a time series (x) using a detrending order (d) that can vary between 1 and 5. As an output this function returns not only the alpha value and the coefficient of determination for the linear fit used to estimate the autosimilarity parameter (alpha), but also the values of window size (n) and Fluctuation (F_n). In order to run the function write in the Command Window of MATLAB the command: [n, F_n, alfa, R2]=DFA_jmk(x,d).

## Additional Information

**How to cite this article:** Guzmán, D. A. *et al.* High resolution, week-long, locomotion time series from Japanese quail in a home-box environment. *Sci. Data* 3:160036 doi: 10.1038/sdata.2016.36 (2016).

## Supplementary Material



## Figures and Tables

**Table 1:** Zone description in the experimental box.

**Zone**	**Description**
1	Feeding compartment with automatic nipple drinker and feeder.
2	Feeding compartment from which the bird has access to the door that connects both compartments.
3	Nesting compartment where a 15×15 cm small rubber nest was placed.
4	Nesting compartment from which the bird has access to the door that connects both compartments.

**Table 2:** Overview of the data files uploaded to FigShare grouped in file sets according to time series type (locomotor, distance ambulated and zone location) recorded from quails in a home-box environment.

**Time series**	**Fileset name**	**Subject**	**Experimental Group**	**Data file names**
**Locomotor**	High resolution locomotor time series in Japanese quail in a home-cage environment over a 6.5 day period (ALL SERIES) (Data Citation 1)	Quail 1	Group 1	Quail_1_group 1.txt
		Quail 2	Group 1	Quail_2_group 1.txt
		⁞	⁞	⁞
		Quail 12	Group 2	Quail_1_group 2.txt
**Distance**	High resolution distance ambulated time series in Japanese quail in a home-cage environment over a 6.5 day period (ALL SERIES) (Data Citation 2)	Quail 1	Group 1	Distance (Quail 1 group 1).txt
		Quail 2	Group 1	Distance (Quail 2 group 1).txt
		⁞	⁞	⁞
		Quail 12	Group 2	Distance (Quail 12 group 2).txt
**Zone**	High resolution zone location time series in Japanese quail in a home-cage environment over a 6.5 day period (ALL SERIES) (Data Citation 3)	Quail 1	Group 1	Zone (Quail 1 group 1).txt
		Quail 2	Group 1	Zone (Quail 2 group 1).txt
		⁞	⁞	⁞
		Quail 12	Group 2	Zone (Quail 12 group 2).txt
